# Composition of Polysaccharides in Hull-Less Barley Sourdough Bread and Their Impact on Physical Properties of Bread

**DOI:** 10.3390/foods12010155

**Published:** 2022-12-28

**Authors:** Sanita Reidzane, Ilze Gramatina, Ruta Galoburda, Vitalijs Komasilovs, Aleksejs Zacepins, Anastassia Bljahhina, Tatjana Kince, Anna Traksmaa, Dace Klava

**Affiliations:** 1 Faculty of Food Technology, Latvia University of Life Sciences and Technologies, Riga Street 22, LV-3004 Jelgava, Latvia; 2 Faculty of Information Technologies, Latvia University of Life Sciences and Technologies, Liela Street 2, LV-3001 Jelgava, Latvia; 3 Center of Food and Fermentation Technologies (TFTAK), Mäealuse 2/4, 12618 Tallinn, Estonia; 4 Department of Chemistry and Biotechnology, Tallinn University of Technology, Akadeemia tee 15, 12618 Tallinn, Estonia

**Keywords:** hull-less barley, hull-less barley sourdough, non-starch polysaccharides, β-glucans, fructans, bioactive compounds

## Abstract

The complex of polysaccharides of the grain transforms during processing and modifies the physical and chemical characteristics of bread. The aim of the research was to characterize the changes of glucans, mannans and fructans in hull-less barley and wholegrain wheat breads fermented with spontaneous hull-less barley sourdough, germinated hull-less barley sourdough and yeast, as well as to analyze the impact of polysaccharides on the physical parameters of bread. By using the barley sourdoughs for wholegrain wheat bread dough fermentation, the specific volume and porosity was reduced; the hardness was not significantly increased, but the content of β-glucans was doubled. Principal component analysis indicates a higher content of β-glucans and a lower content of starch, total glucans, fructans and mannans for hull-less barley breads, but wholegrain wheat breads fermented with sourdoughs have a higher amount of starch, total glucans, fructans and mannans, and a lower content of β-glucans. The composition of polysaccharides was affected by the type of flour and fermentation method used.

## 1. Introduction

Barley (*Hordeum vulgare* L.) has been cultivated for centuries all over the world, thanks to its diversity and ability to adapt to the different conditions of climate and soil [1,2,3]. In the Northern European regions and countries around the Baltic Sea, barley has been grown historically and has been used to bake wholegrain bread of unspecific form. It was viewed as a source of strength and health. The hull-less barley varieties have been acknowledged as a highly valuable raw material for nutrition, containing unique and balanced nutrient compositions with a high content of proteins, starch, and non-starch polysaccharides [3,4].

Depending on the type of grain, flour contains polysaccharides of multiple structures and varied biological activity. The natural sources of polysaccharides are plants and microorganisms [5]. The non-starch polysaccharides, such as β-glucans, mannans and fructans are named bioactive compounds because they exhibit beneficial health effects and prevent diseases [6,7,8]. The bran, the husk and cereal endosperm cell walls, as well as the aleurone layer contain non-starch polysaccharides, which are the most important fiber components [9,10,11]. The endosperm cell walls of barley are rich in mixed linkage β-glucans. Wheat cell walls have low contents of β-glucans found in bran [12]. In a water solution, β-glucans create viscous solutions, therefore the use of barley flour in baking has been limited. Other cell wall components are water-soluble mannans that are classified as dietary fibers; they have high viscosity and low palatability [13]. The latest research showed that of all cell wall polysaccharides, mannans make up 4–7% [10]. Fructans (linear or branched polymers) are non-digestible carbohydrates classified as dietary fibers, which are found in plant cell walls and microbial environments [14]. Fructans influence human health in two different ways [15]. For humans with functional gastrointestinal disorders like irritable bowel syndrome, the fructan polysaccharides can cause bloating and stomach pain. However, the beneficial or prebiotic effects of fructans are related to the bacterial transformation to the short chain fatty acids [16]. These polysaccharides influence the texture of bread, which is dependent on their solubility and viscosity characteristics [17].

Barley flour is mainly used to enrich wheat flour with dietary fibers, especially with biologically active β-glucans. However, depending on the used technology, the optimal amount of barley flour to replace wheat flour is 20–40%, so that the product would keep the quality to an acceptable level, as barley flour has a negative effect on the texture and volume of crumb [18]. Barley flour can be used both for bread-baking and in the preparation of sourdough, as it is a rich substrate for the creation of a microbial community. Traditionally, wheat and rye sourdoughs are used for bread-baking, but barley and also germinated barley can be used as an alternative raw material to make sourdough [19,20,21,22]. Germination increases the enzymatic activity of the grains. During the last decades, many researchers have been exploring the fermentation of sourdough and its benefits [23,24]. The fermentation provides the wholegrain bread with unique taste characteristics, improves the nutrition quality and prolongs the shelf life. In the sourdough and dough fermentation the complex of polysaccharides is modified, therefore it impacts the technological functionality and nutritional value. The metabolic activity of microorganisms causes the biochemical modifications of sourdough. During the fermentation of sourdough, the lactic acid bacteria can, using sucrose, synthesize glucan and fructan-type exopolysaccharides [25,26]. These polymers act as hydrocolloids, and their impact in dough and bread is based on the water-binding capacity in the dough preparation stage and the mutual interaction with other components of dough, like starch and proteins [27]. In addition to biochemical interactions, there are also chemical transformation in nutrients during sourdough and dough fermentation. This is achieved by modifying the nutritional complex of compounds of the flour and impacting the technological properties of bread [28].

The goal of this study was to characterize the changes of glucans, fructans and mannans in hull-less barley and wholegrain wheat bread fermented with spontaneous hull-less barley, germinated barley sourdough and yeast, as well as to analyze the impact of these polysaccharides on the physical attributes of breads.

## 2. Materials and Methods

### 2.1. Materials

The hull-less barley (moisture 12.1 ± 0.4%, protein 17.8 ± 0.3%) (*Hordeum vulgare* L.) of the Kornelija grain variety was obtained from Stende Research Center (Institute of Agricultural Resources and Economics, Latvia). Wholegrain wheat flour was purchased from AS Rigas dzirnavnieks (Riga, Latvia).

The following materials were used in the preparation of the doughs: fresh yeast purchased from Lesaffre SA (Wolczyn, Poland), sugar (Nordzucker Polska S.A., Opalenica Poland), salt (Artemsil, Soledar, Ukraine), barley malt of moisture 9%, diastatic power 250 WK (Latmalt SIA, Jelgava, Latvia).

The research was carried out in the scientific laboratories of the Faculty of Food Technology of the Latvia University of Life Sciences and Technologies.

Before the germination or sprouting, grains were rinsed and soaked in water at 22 °C for 24 h. The germination was performed in a climatic chamber (Memmert HPP110, Schwabach, Germany) at 35 ± 1 °C, maintaining a constant relative air humidity of 80%. Germination time was up to 24 h. Germinated grains were dried up to 4 h in the universal oven (Memmert UF55, Schwabach, Germany) at 60 ± 1 °C [29].

Hull-less barley (HB) and germinated hull-less barley (HB-G) grains were milled using a Hawos laboratory mill (Hawos Kornmühlen GmbH, Hamburg, Germany). Obtained flour (HBF; HBF-G) was sieved through AS 200 sieves (Retsch GmbH, Haan, Germany). Resulting flour particle size was 160–710 µm, of which 50% was on the sieve 450 µm, 28% was on the sieve 315 µm and 10% above 710 µm.

### 2.2. Spontaneous Hull-Less Barley and Germinated Hull-Less Barley Sourdough Preparation

Spontaneous hull-less barley sourdough (HBS-S) and spontaneous germinated hull-less barley sourdough (HBS-G) were prepared under laboratory-controlled conditions. For the preparation of spontaneous sourdough, the three-stage procedure was used. The preparation of HBS-S was done at optimal fermentation conditions established in previous studies [30] and described in Table 1. To start the fermentation, 100 g HB flour and 113 mL of water was mixed. For the second and third step, 100 g of previously fermented sourdough (inoculum) was added, then mixed with the new amount of flour and water specified in Table 1. The same procedure was done for the preparation of HBS-G.

Characterization of sourdoughs: for HBS-S—lactic acid bacteria 8.0 log CFU (colony forming units)/g; yeast ca 7.0 log CFU/g, pH 3.8, for HBS-G—lactic acid bacteria ca 8.0 log CFU/g; yeasts ca 6.5 log CFU/g, pH 3.9.

### 2.3. Bread Making

Hull-less barley breads (HBB) and wholegrain wheat breads (WWB) were prepared in three different ways: fermented with spontaneous hull-less barley sourdough (HBB-S and WWB-S), fermented with spontaneous germinated hull-less barley sourdough (HBB-G and WWB-G) and with yeast fermentation (HBB-Y and WWB-Y).

For sourdough bread (HBB-S, HBB-G and WWB-S, WWB-G) preparation flour, spontaneous sourdough (the flour content of sourdough was 16% of total flour weight), salt, sugar, barley malt and water were used. For bread with yeast fermentation (HBB-Y and WWB-Y) flour, yeast, salt, sugar, barley malt and water were added. Composition of ingredients of HBB and WWB was according to the recipe in Table 2. The dough components were mixed in a dough mixer (Kenwood, Hampshire, UK) for 5 min on slow, then dough proofed for 30 min and divided into 400 g pieces with wet hands. The doughs were fermented in the molds for 4 h at 30 °C in a proofer (Sveba Dahlen AB, Fristadt, Sweden). The samples were baked for 20 min at 190 °C with steam 3 s. Two separate batches of each bread type were prepared.

### 2.4. Determination of Polysaccharides

The samples of each bread were lyophilized in a freeze-dryer FT33 (Armfield Ltd., Hampshire, UK) maintaining −40 °C and 6.4 Pa in a condenser chamber for 72 h. Lyophilized samples were milled using a Foss Knifetec 1095 Mill (FOSS Analytical AB, Hoganas Sweden).

The content of the total glucans and mannans was measured from flours and from lyophilized bread samples using a previously published method [31] with modifications. The concentrations of carbohydrates were determined by high-performance liquid chromatography (HPLC, Alliance 2795 system, Waters, Milford, MA, USA) using a BioRad HPX—87C column (Hercules, CA, USA) held at 85 °C with isocratic elution of ultrapure water at a flow rate of 0.6 mL/min. Refractive index (RI) (model 2414; Waters, Milford, MA, USA) detector was used for detection of the substances. Arabinose was used as an internal standard for glucose quantification. The quantification of total glucans and mannans were determined using equation (1), where C (mg/g)—the concentration of glucose/ mannose liberated after hydrolysis, 180 g/mol—molecular mass (g/mol) of given hexose; 162 g/mol—molecular mass (g/mol) of given hexose without H_2_O; 1000—converts mg to g; 100—converts to %.

C % = [(C /1000 / 180 g/mol) × 162 g/mol] × dilution factor × 100(1)

Starch analysis was performed in accordance with EN ISO 10520:2001. The content of β-glucans was determined according to the ICC Standard Method No. 168 using Assay kit (Megazyme Ltd., Wicklow, Ireland). Total dietary fiber (TDF) was determined according to the AOAC 985.29.

Fructans were determined in lyophilized samples using Megazyme kit K-FRUC (Megazyme Ltd., Wicklow, Ireland) at 410 nm (UV-VIS spectrophotometer, Thermo electron Corp., Rugby, UK).

### 2.5. Wholegrain Barley and Wheat Sourdough Bread Technological Characteristics

The volume of breads was determined by rapeseed displacement according to the AACC Method 10-05.01 [32]. Specific volume was calculated as loaf volume and bread weight ratio.

Bread porosity was determined using Zhuravlev device (Biomer Ltd., Krasnoobsk, Russia) according to a previously described method by Cizeikiene et al. [33].

Analysis of bread texture was performed by means of a TA.HD.Plus texture analyzer (Stable Microsystems, Godalming, UK) according to AACC 74-09 with some modifications [34]. Trigger force was set at 0.049 N. Two slices of mechanically sliced bread (7 mm thick) were compressed with 25 mm aluminum cylindrical probe at 1.7 mm/s speed. The original software Texture Exponent 32 (Stable Microsystems, Godalming, UK) allowed the measurement of peak positive force (N), which describes hardness and peak negative force (N) describing bread stickiness. Analysis was performed 24 h after baking. The results are means of ten replicates.

### 2.6. Statistical Analysis

The chemical and physical parameters of HBB and WWB breads were subject to statistical analysis. Statistically significant differences (*p* < 0.05) in chemical and physical parameters of the samples were determined using pairwise Tukey’s honest significant difference (HSD) test.

The principal component analysis (PCA) and correlation analysis were used to determine relationships between chemical and physical parameters and breads samples. Data transformation, analysis and visualization were performed on a custom processing pipeline built using open source Python 3.8 and its libraries NumPy [35], Pandas [36], Statsmodels [37], and Scikit-learn [38].

## 3. Results and Discussion

### 3.1. Starch, Total Dietary Fiber and Non-Starch Polysaccharides Composition in Flours and Breads

#### 3.1.1. The Content of Starch and Total Glucans

The original complex of polysaccharides of the grain changes during processing. This is due to different mechanical processes, hydrolysis and bioprocessing, such as germination and fermentation, therefore it has an effect on the quality of bread in different ways. Significant changes of these substances happen as a result of the activation of the endogenous enzymatic system of the flour, as well as the influence of biochemical transformations caused by microorganisms during fermentation [39].

**Figure 1:**
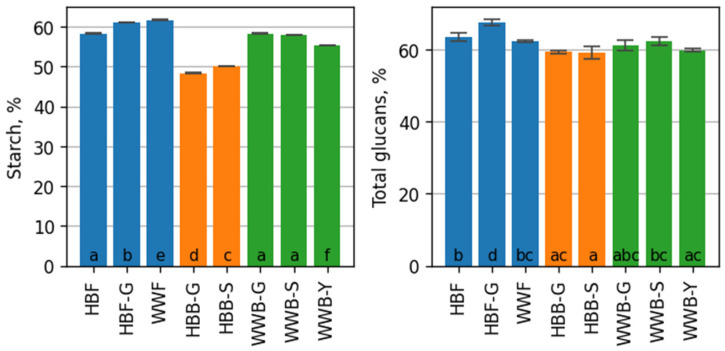
The content of starch and total glucans, % of dry weight of the flours (in blue): hull-less barley flour (HBF), germinated hull-less barley flour (HBF-G) and wholegrain wheat flour (WWF); of the hull-less barley breads (in orange): fermented with germinated hull-less barley sourdough (HBB-G); fermented with hull-less barley spontaneous sourdough (HBB-S); of the wholegrain wheat breads (in green): fermented with spontaneous hull-less barley sourdough (WWB-S); fermented with germinated hull-less barley sourdough (WWB-G); wholegrain wheat bread with yeast fermentation (WWB-Y). Each result represents mean ± standard deviation (*n* = 3). Values with the different letters are significantly different based on the Tukey’s HSD test.

Starch and non-starch glucan type polysaccharides, mainly β-glucans, are included in the total complex of glucan compounds [5,9]. In cereal, starch makes up the majority of the polysaccharides in grains, ranging between 50% and 70% [17,40]. As seen in Figure 1, the content of starch in WWF was determined by 61.68 ± 0.14% of dry weight (dw). It differed significantly from the content of starch in HBF—58.27 ± 0.11% dw and in HBF-G—61.0 ± 0.07% dw. After 24 h of barley grain germination, there were no observations of reduced starch content in the flour from germinated hull-less barley; this fact is confirmed by other researches on the influence of germination time on changes in starch content. Previous research showed that by increasing fermentation time of the grains, the content of starch was decreased, and the cell walls in the process of hydrolysis were decomposed by hydrolyzing enzymes [41,42]. Significant changes of starch occurred during the fermentation of sourdough and dough, under the influence of endogenous α-amylase and β-amylase. The results (Figure 1) show that in HBB-S and HBB-G bread, the content of starch is significantly reduced compared to its content in flour. Sourdough fermentation is related with starch hydrolysis and reduced digestibility [43,44,45]. The highest decrease of starch content, about 17.1% dw, was observed in the bread HBB-G, which signified the high enzymatic activity of the germinated hull-less barley flour. Starch plays a key role in establishing crumb texture. Starch content was significantly higher in WWB breads compared to HHB breads. The chemical composition of HBB-Y was not analyzed due to unacceptable physical characteristics.

In the result of the metabolic activity of lactic acid bacteria in sourdough and dough, there is a possibility that the microbial glucan type exopolysaccharides can also be produced with different structures and characteristics. The β-glucans of grains and the microbial glucan type exopolysaccharides influence the technological properties and nutritional value of bread, because they potentially act as hydrocolloids and prebiotics [21,26,46]. The total content of glucans was reduced slightly by 6–7% in HBB-G and HBB-S bread compared to HBF. The starch part of the total glucans was reduced in HBB-G and HBB-S bread, in comparison to the corresponding part in barley flour. The highest total glucans content was observed in WWB-S. In the samples HBB-G and WWB-Y there were no significant differences in the total glucans content.

#### 3.1.2. Total Dietary Fiber and Non-Starch Polysaccharides Composition

**Figure 2:**
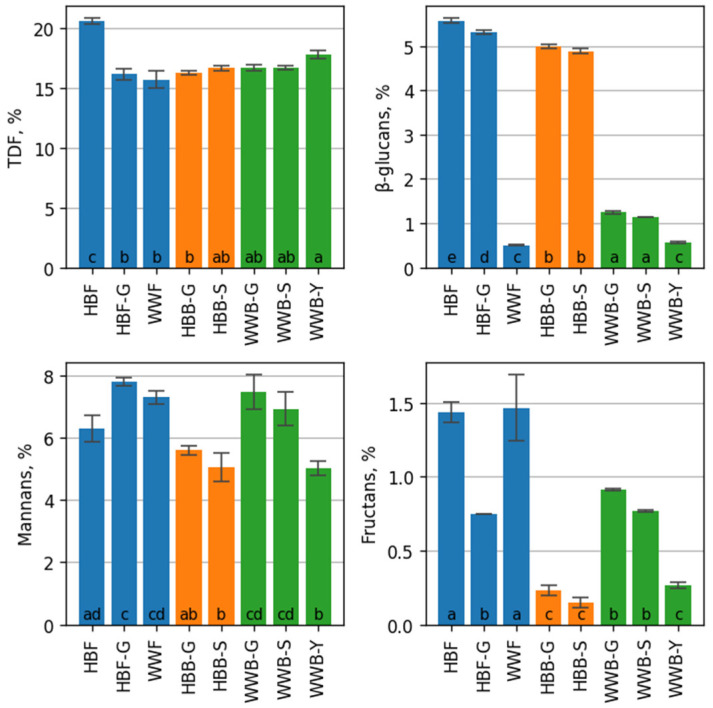
TDF and non-starch polysaccharides composition, % of dry weight, of the flours (in blue): hull-less barley flour (HBF), germinated hull-less barley flour (HBF-G) and wholegrain wheat flour (WWF); of the hull-less barley breads (in orange): fermented with germinated hull-less barley sourdough (HBB-G); fermented with hull-less barley spontaneous sourdough (HBB-S); of the wholegrain wheat breads (in green): fermented with spontaneous hull-less barley sourdough (WWB-S); fermented with germinated hull-less barley sourdough (WWB-G); wholegrain wheat bread with yeast fermentation (WWB-Y). Each result represents mean ± standard deviation (*n* = 3). Values with the different letters are significantly different based on the Tukey’s HSD test.

TDF content in different varieties of hull-less barley can be between 11% and 20% [3]. In wholegrain wheat, the content of TDF can be between 11% and 17%, which is concentrated in the bran part. The results of the current experiment (Figure 2) showed that the highest content of TDF was in HBF—20.55 ± 0.25% dw, but in HBF-G and WWF flour it was significantly lower 16.14 ± 0.46% dw and 15.67 ± 0.73% dw, respectively. In HBB-S, HBB-G bread, TDF had significantly decreased compared to its content in the HBF. In HBB-S, WWB-S, WWB-G bread, a significant difference in TDF was not found. The highest decrease in the content of TDF was observed in HBB-G—21%. The increase in solubility of non-starch polysaccharides during the process of dough fermentation explains the observed decrease in TDF. Researchers reason that the increase in TDF was with the generation of resistant starch during baking [47]. A slight increase was found in WWB-Y. Among the bread samples, the highest TDF content was in WWB-Y. The TDF content of breads HBB-S, WWB-G, WWB-S was not significantly different.

In different barley species, the content of β-glucans varied between 4 and 9% [3], but in wholegrain wheat flour it could be 0.3 ± 3.0% [6]. The unique property of barley grains is that β-glucans are evenly spread in the kernel, and the removal of outer layers does not significantly influence the amount of β-glucans [4]. HBF contained 5.57 ± 0.05% dw of β-glucans. In HBF-G, the content of β-glucans had decreased slightly to 5.31 ± 0.04% dw compared to its content in HBF. In wholegrain wheat bread WWB-Y, the content of β-glucans was 0.56 ± 0.01% dw, and it was two-fold less than in WWB-S and WWB-G bread and eight times less than in HBB-S and HBB-G. The adding of hull-less barley sourdough significantly increased the content of β-glucan in wholegrain wheat WWB-S and WWB-G bread, up to 1.14 ± 0.01% dw and 1.24 ± 0.02% dw, respectively. A slight decrease in β-glucans content was observed in hull-less barley bread HBB-S and HBB-G, compared to flour HBF. Thus, the results determined in the present study are in a good correspondence with the previously reported results [47,48]. During mixing, fermentation and proofing, degradation of β-glucans happens due to endogenous β-glucanase of wholegrain wheat and barley flour [39]. The method of bread preparation has a great influence on the final content of β-glucans in the bread.

The content of mannans in WWF flour was 7.29 ± 0.20% dw. In hull-less barley flour (HBF) the content of mannans was 6.29 ± 0.41% dw. In the samples of hull-less barley bread HBB-S and HBB-G, the content of mannans had decreased until 5.23 ± 0.36% and 5.24 ± 0.61% dw, respectively. In the samples of wholegrain wheat bread WWB-S and WWB-G, the content of mannans was not significantly different from flour, 6.92 ± 0.55% and 7.45 ± 0.56% dw, respectively. However, in the sample WWB-Y, the content of mannans was the lowest—5.01 ± 0.24% dw. Between the samples WWB-S and WWB-G, there was no significant difference in the content of mannans. The highest content of mannans was in WWB-S, WWB-G and it was significantly higher than in HBB-S, HBB-G and WWB-Y.

The highest content of fructans was found in wholegrain wheat flour (WWF)—1.47 ± 0.22% dw. It did not significantly differ from the content in hull-less barley flour—1.44 0.07% dw. Ispiryan and co-workers (2020) showed similar results of the content of fructans in wheat and barley flour [15]. The amount of fructans in barley grain can be 0.9–4.2% dw depending on the variety of barley and the growth environment [49], and in different types of wheat it could be 0.7–2.9% dw [6]. In germinated hull-less barley flour (HB-G) the amount of fructans was 0.75

± 0.00%, and this was significantly lower than in hull-less barley flour (HBF). Due to the increased enzymatic activity, the amount of fructans could either be synthesized or degraded [50,51,52]. The nature of changes depends on factors such as germination time and temperature that impacts the germination [50].

Results (Figure 2) showed the highest content of fructans in the samples of wholegrain wheat sourdough breads: WWB-S and WWB-G, but in HBB-S, HBB-G and WWB-Y the fructans content was significantly lower. Of all WWB, in bread WWB-Y, the content of fructans had a significantly lower value —0.27 ± 0.02% dw. Fructan degradation is affected by enzyme invertase, secreted by yeast *Saccharomyces cerevisiae* cells during fermentation [16,53]. A longer dough fermentation time significantly reduces the level of fermentable oligosaccharides, disaccharides, monosaccharides and polyols (FODMAP) up to 90% [6,15,50]. The largest decrease of fructans of 89.6% was observed in the barley bread HBB-S, as well as in wholegrain wheat bread WWB-Y—81.6%, which is related to the activation of endogenous enzymes at the lower pH and bacterial hydrolytic enzyme activity [50].

The behavior of b-glucans, fructans and mannans is similar in barley breads, indicating a decrease of content during the processing of breads.

### 3.2. Physical Characteristics of Hull-Less Barley and Wholegrain Wheat Breads

**Figure 3:**
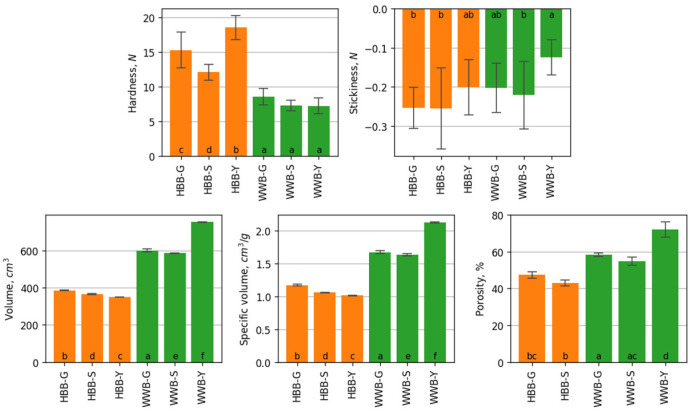
Physical characteristics of the hull-less barley bread (in orange): fermented with spontaneous germinated hull-less barley sourdough (HBB-G); fermented with hull-less barley spontaneous sourdough (HBB-S); with yeast fermentation (HBB-Y); of the wholegrain wheat bread (in green): fermented with spontaneous hull-less barley sourdough (WWB-S); fermented with spontaneous germinated hull-less barley sourdough (WWB-G); with yeast fermentation (WWB-Y). Each result represents mean ± standard deviation (*n* = 6). Values with the different letters are significantly different based on the Tukey’s HSD test. For sample HBB-Y, porosity was not determined.

As a result of flour processing the complex of polysaccharides changes, and it influences the technological properties of bread. The results of crumb hardness, stickiness, volume, specific volume and porosity of breads are presented in Figure 3. The hardness of sourdough wheat bread WWB-S and WWB-G was 7.35 ± 0.75 N and 8.56 ± 1.13 N, respectively. The whole-grain wheat bread with yeast fermentation (WWB-Y) had the lowest hardness: 7.2 ± 1.09 N, however, it did not significantly differ from the hardness of wholegrain wheat bread WWB-S and WWB-G. Results show that germinated barley sourdough increased the hardness of the crumb of hull-less barley and wheat bread: HBB-G and WWB-G, and this fact corresponds to the researches done by other scientists [54,55]. Barley bread HBB-G, HBB-S and HBB-Y had a higher hardness compared to wheat breads WWB-G, WWB-S and WWB-Y. The lowest stickiness was observed in WWB-Y. In the samples of HBB-G, HBB-S and WWB-S, stickiness was not significantly different.

The specific volume was the lowest in the hull-less barley bread HBB-S and HBB-G. The volume and the specific volume of WWB-Y was significantly higher than that of wheat and barley breads with sourdough. In this research, the preparation of barley bread HBB-Y was considered unacceptable because of the highly dense texture without the increase in volume, and without the signs of yeast metabolism. It was not possible to determine the porosity of HBB-Y. The specific volume was similar for both WWB-S and WWB-G breads, and HBB-S and HBB-G breads. However, other scientists have indicated the highest porosity and specific volume of wholegrain wheat bread by using wholegrain wheat spontaneous sourdough [33]. The highest porosity (71.73 ± 0.70%) was observed in WWB-Y, which showed the intensive activity of yeasts and release of CO_2_. The scientists have mentioned that fructans are the source of fermented sugars, and the products of their hydrolysis forms a significant part of CO_2_ in the first stages of dough fermentation, which could also influence the porosity of bread [6,53]. The high content of dietary fiber in the wholegrain wheat flour weakens the gluten network, increases the hardness of the crumb, reduces the loaf volume and modifies the porosity compared to white bread [6].

In breads WWB-S and WWB-G fermented with sourdough, the porosity was higher than that observed in HBB-S and HBB-G. Adding the germinated barley sourdough resulted in the higher porosity and larger volume both in barley and wheat breads, which can be explained by an increase of enzymatic activity during the time of germination. This, in turn, impacts the increase of starch degradation and yeast metabolism, and the release of CO_2_ [54,55].

**Figure 4:**
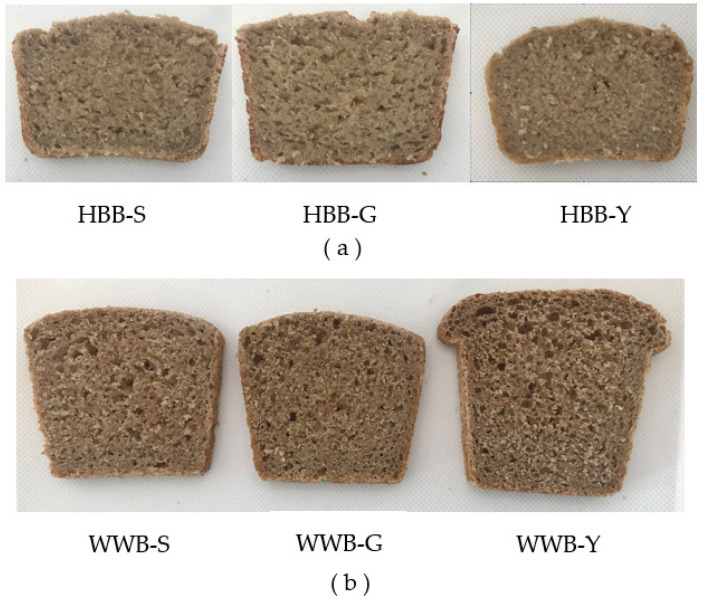
The crumb (**a**) of the hull-less barley bread fermented with hull-less barley spontaneous sourdough (HBB-S); of the hull-less barley bread fermented with spontaneous germinated hull-less barley sourdough (HBB-G); and of the hull-less barley bread with yeast fermentation (HBB-Y) and wholegrain wheat breads; (**b**) of the wholegrain wheat bread fermented with spontaneous hull-less barley sourdough (WWB-S) and of the wholegrain wheat bread fermented with spontaneous germinated hull-less barley sourdough (WWB-G) and wholegrain wheat bread with yeast fermentation (WWB-Y).

In the spontaneous sourdough hull-less barley breads, the crumb was less porous and more compact (Figure 4a) in comparison to wholegrain wheat bread (Figure 4b). Mexican and Turkish researchers have pointed out the compact texture of barley bread and the prolonged irregular pores of the crumb [56,57]. Unlike the wholegrain wheat bread, in bread made from hull-less barley flour, the structure is mainly made by a network of starch and non-starch polysaccharides.

The wholegrain wheat bread with yeast fermentation showed the best technological characteristics—crumb hardness, specific volume and porosity. However, it had the lowest content of soluble non-starch polysaccharides such as β-glucans, mannans and fructans, but with the highest total dietary fiber content. The use of spontaneous barley sourdough for wholegrain wheat bread dough fermentation reduced the specific volume and porosity, but did not change the hardness significantly. However, the content of β-glucans was significantly increased. For barley breads HBB-S and HBB-G, the technological characteristics were similar, and there were no significant differences in the content of non-starch polysaccharides.

### 3.3. Relationship between Physical and Chemical Parameters of Breads

**Figure 5:**
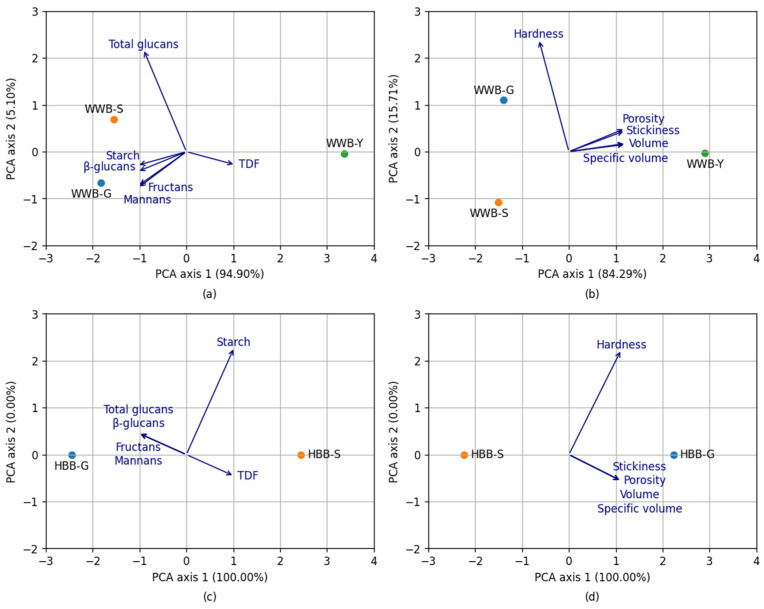
(**a**,**c**)—Principal component analysis (PCA) between chemical parameters (starch, total dietary fiber (TDF), total glucans, β-glucans, fructans, mannans) and hull-less barley bread (HBB-S; HBB-G) or wholegrain wheat bread (WWB-S; WWB-G; WWB-Y), (**b**,**d**)—PCA between physical parameters (volume, specific volume, hardness, stickiness, porosity) and hull-less barley bread (HBB-S; HBB-G) or wholegrain wheat bread (WWB-S; WWB-G; WWB-Y).

Principal component analysis (PCA) was performed to determine the relationship between the chemical parameters and physical parameters of WWB (Figure 5a,b) and HBB (Figure 5c,d). The relationships for all measured data were visualized in biplots. The samples of WWB-G were associated with a higher content of starch and non-starch polysaccharides separate from WWB-S, but WWB-Y—by a higher content of TDF. PCA axis 1 (Figure 5c) indicates that HBB-S was characterized by higher starch and TDF content, but HBB-G by higher total glucans and non-starch polysaccharides. In turn, the physical characteristics (Figure 5b) of wholegrain wheat breads were located in different zones of the plot. WWB-G was associated with a higher hardness (Figure 5d).

**Figure 6:**
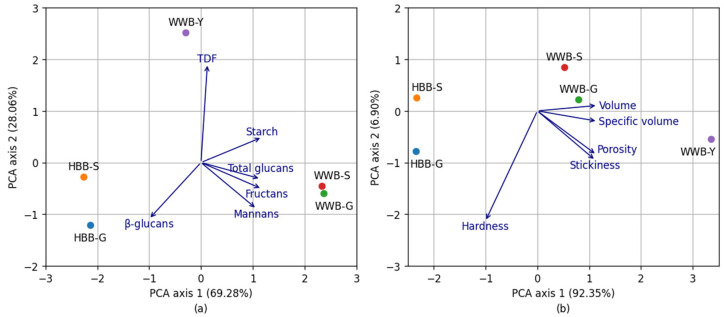
(**a**)—Principal component analysis (PCA) between chemical parameters (starch, total dietary fiber (TDF), total glucans (glucans), β-glucans, fructans, mannans) and hull-less barley bread (HBB-S; HBB-G) and wholegrain wheat bread (WWB-S; WWB-G; WWB-Y), (**b**)—PCA between physical parameters (volume, specific volume, hardness, stickiness, porosity) and hull-less barley bread (HBB-S; HBB-G) and wholegrain wheat bread (WWB-S; WWB-G; WWB-Y).

PCA differentiated between HBB and WWB according to chemical (Figure 6a) and physical (Figure 6b) parameters. The samples of breads were grouped in different zones of plot/plain. The first two principal components explain 97.34% of variance of chemical parameters of hull-less barley breads and wholegrain wheat breads. PCA axis 1 (69.28%) indicates a higher content of β-glucans and lower content of starch, glucans, fructans and mannans for HBB-S and HBB-G, but WWB-S; WWB-G have higher amount of starch, glucans, fructans and mannans, and lower content of β-glucans. PCA axis 2 (28.06%) shows that characteristics of WWB-Y differ to those of sourdough bread. In this case, bread prepared with yeast technologies is characterized by a higher amount of TDF, and a lower number of other elements. Figure 6 b shows that the two principal components explain 99.25% of the total variance. PCA axis 1 (92.35%) indicate that HBB-S and HBB-G have a higher hardness and lower volume, specific volume and porosity compared to WWB-Y.

**Figure 7:**
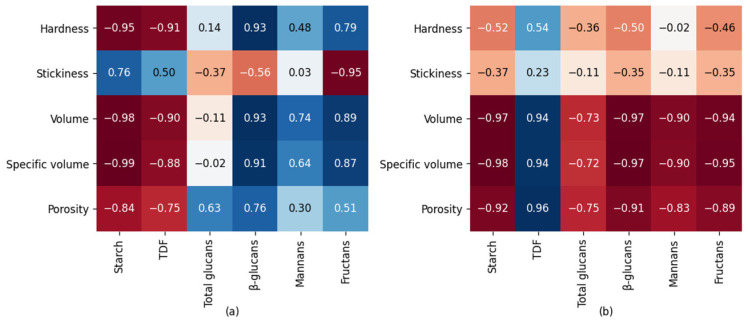
Correlation between chemical and physical characteristics of HBB (**a**) and WWB (**b**) breads.

Correlation analysis between chemical and physical characteristics of HBB and WWB breads is shown in Figure 7. A very high positive correlation for HBB was observed for β-glucans and hardness, volume, and specific volume, but it was not significant. Significant negative correlations of WWB were observed between starch, β-glucans and volume, specific volume and porosity. There was a significant positive correlation between TDF and the porosity parameters in WWB.

## 4. Conclusions

Spontaneous hull-less barley sourdough and spontaneous germinated hull-less barley sourdough can be used to prepare barley and wholegrain wheat bread. From the viewpoint of bioactive polysaccharides, the composition of bread fermented with spontaneous hull-less barley sourdough was similar to the composition of bread fermented with spontaneous germinated barley sourdough. The method of fermentation influenced the content of fructans and β-glucans in bread. In the wholegrain wheat bread with yeast fermentation, the content of fructans decreased faster in comparison to fermentation with barley sourdough. When using the barley sourdough, the content of β-glucans in the wholegrain wheat bread increased twice, but nevertheless it was lower than in the barley sourdough bread. The wholegrain wheat sourdough bread was characterized by a higher content of fructans and mannans, and the same amount of total dietary fiber as in barley sourdough bread.

The use of barley sourdough improved the volume, porosity, and reduced hardness of barley bread. Bread made with germinated barley sourdough had a higher hardness and porosity compared to bread with spontaneous barley sourdough. Spontaneous hull-less barley sourdough and germinated hull-less barley sourdough had a negative effect on the physical properties of breads. In comparison to wholegrain wheat bread prepared with yeast, wholegrain wheat breads fermented with sourdough had a higher content of polysaccharides.

## Figures and Tables

**Table 1:** Spontaneous hull-less barley sourdough (HBS-S) and spontaneous germinated hull-less barley sourdough (HBS-G) composition and preparation parameters ^1^.

Stage	Sourdough Composition			Calculated Flour Amount (g/100g) in Inoculum	Calculated Flour Amount (%) in Sourdough	Parameters	
	Inoculum (g)	Flour (g)	Water (g)			Temperature (°C)	Time (h)
1st		100	113		43.5	30	24
2nd	100	100	137	43.5	42.5	31	14
3rd	100	100	130	42.5	43.2	28.5	12

^1^ Adapted from our previous study [30] with some modifications for best leavening capacity.

**Table 2:** The recipe of hull-less barley bread (HBB) and wholegrain wheat (WWB) bread making. Hull-less barley flour (HBF); germinated hull-less barley flour (HBF-G); wholegrain wheat flour (WWF); hull-less barley bread fermented with hull-less barley spontaneous sourdough (HBB-S); hull-less barley bread fermented with germinated hull-less barley sourdough (HBB-G); wholegrain wheat bread fermented with spontaneous hull-less barley sourdough (WWB-S); wholegrain wheat bread fermented with germinated hull-less barley sourdough (WWB-G); wholegrain wheat bread with yeast fermentation (WWB-Y).

Materials	HBB			WWB		
	Spontaneous Sourdough Fermentation	Spontaneous Sourdough Fermentation	Yeast Fermentation	Spontaneous Sourdough Fermentation	Spontaneous Sourdough Fermentation	Yeast Fermentation
	HBB-S % fw	HBB-G % fw	HBB-Y % fw	WWB-S % fw	WWB-G % fw	WWB-Y % fw
HBF	84.0	84.0	100.0			
WWF				84.0	84.0	100.0
HBS-S	37.0			37.0		
HBS-G		37.0			37.0	
Barley malt	3.0	3.0	3.0	3.0	3.0	3.0
Salt	1.5	1.5	1.5	1.5	1.5	1.5
Sugar	1.5	1.5	1.5	1.5	1.5	1.5
Fresh yeast			3.0			3.0
Water	67	67	67	67	67	67

% fw—% of total flour weight. For abbreviations different designations were used: -S for spontaneous hull-less barley sourdough, -G for spontaneous germinated hull-less barley sourdough and -Y for yeast fermentation.

## Data Availability

The data used to support the findings of this study can be made available by the corresponding author upon request.
